# Functional Characterization of Endophytic Fungal Community Associated with *Oryza sativa* L. and *Zea mays* L.

**DOI:** 10.3389/fmicb.2017.00325

**Published:** 2017-03-02

**Authors:** Momota Potshangbam, S. Indira Devi, Dinabandhu Sahoo, Gary A. Strobel

**Affiliations:** ^1^Department of Biotechnology, Microbial Resources Division, Institute of Bioresources and Sustainable DevelopmentImphal, India; ^2^Department of Plant Sciences and Plant Pathology, College of Agriculture, Montana State UniversityBozeman, MT, USA

**Keywords:** fungal endophyte, stress tolerance, plant growth, biocontrol agent, phytopathogens

## Abstract

In a natural ecosystem, the plant is in a symbiotic relationship with beneficial endophytes contributing huge impact on its host plant. Therefore, exploring beneficial endophytes and understanding its interaction is a prospective area of research. The present work aims to characterize the fungal endophytic communities associated with healthy maize and rice plants and to study the deterministic factors influencing plant growth and biocontrol properties against phytopathogens, *viz, Pythium ultimum, Sclerotium oryzae, Rhizoctonia solani*, and *Pyricularia oryzae*. A total of 123 endophytic fungi was isolated using the culture-dependent approach from different tissue parts of the plant. Most dominating fungal endophyte associated with both the crops belong to genus *Fusarium, Sarocladium, Aspergillus*, and *Penicillium* and their occurrence was not tissue specific. The isolates were screened for *in vitro* plant growth promotion, stress tolerance, disease suppressive mechanisms and based on the results, each culture from both the cereal crops was selected for further study. *Acremonium* sp. (ENF 31) and *Penicillium simplicisssum* (ENF22), isolated from maize and rice respectively could potentially inhibit the growth of all the tested pathogens with 46.47 ± 0.16 mm to 60.09 ± 0.04 mm range zone of inhibition for ENF31 and 35.48 ± 0.14 to 62.29 ± 0.15 mm for ENF22. Both significantly produce the defensive enzymes, ENF31 could tolerate a wide range of pH from 2 to 12, very important criteria, for studying plant growth in different soil types, especially acidic as it is widely prevalent here, making more land unsuitable for cultivation. ENF22 grows in pH range 3–12, with 10% salt tolerating ability, another factor of consideration. Study of root colonization during 7th to 30th days of growth phase reveals that ENF31 could colonize pleasantly in rice, though a maize origin, ranging from 1.02 to 1.21 log10 CFU/g root and in maize, it steadily colonizes ranging from 0.95 to 1.18 log10 CFU, while ENF22 could colonize from 0.98 to 1.24 Log10CFU/g root in rice and 1.01 to 1.24Log10CFU/g root in maize, just the reverse observed in *Acremonium* sp. Therefore, both the organism has the potency of a promising Bio-resource agent, that we must definitely explore to fill the gap in the agriculture industry.

## Introduction

Plants are in continuous interaction with microbes, some turning out to be pathogens and some beneficial to the host. There are several studies reporting the beneficial aspect of certain groups of microbes termed as the endophytes (González-Teuber, 2016; Khan et al., 2016). Endophyte is defined as an important group of widespread and diverse plant symbionts that live asymptomatically and sometimes systematically within plant tissues without causing symptoms of disease (Promputtha et al., 2005; Porras-Alfaro and Bayman, 2011). Often reported as a less unexplored area of study, especially in developing countries, as they say, a largely hidden component of fungal biodiversity (Arnold, 2007; Rodriguez et al., 2009). Endophytic fungi represent an important and quantifiable component of fungal biodiversity in plants that impinge on plant community diversity and structure (Higgins et al., 2007; Krings et al., 2007; Jumpponen and Jones, 2009; Porras-Alfaro and Bayman, 2011). Vogl first isolated and cultured asymtomless endophytes from seeds of *Lolium temulentum* (Vogl, 1898). Research and knowledge of endophytic fungi are just an introduction needing added data and intense study to prove a hidden Bioresource. Nonsystemic endophytic fungi identified in a wide range of host plant species have met enormous attention because of their striking species diversity and different ecological niches (Rodriguez et al., 2008). Fungal endophytes are drawing attention to different area of researchers because of the unrevealing benefits it endows to the host in different ways, such as producing bioactive secondary metabolites, promoting growth, good yield, inducing host plants to tolerate both biotic and abiotic stresses and disease resistance, a highly desirable crop trait in the sustainable agricultural industry (Jose et al., 2009; Le et al., 2009; Hartley et al., 2015; Amin, 2016). Endophytes emerge as defensive Bioresources, with a great potential application that encourages researchers to focus more on its study and the mechanism by which it protects the associated host. In an investigative study performed (Waqas et al., 2015) on sunflower plants induced with endophytic fungi *Penicillium citrinum* and *Aspergillus terreus*, the plants showed disease resistance against *Sclerotium rolfsii* and overall improved the biomass yield of sunflower plants. Another demonstrative study performed on rice plants against cold, salt and drought stress concluded that rice plant can exhibit stress via symbiosis with class 2 fungal endophytes, proving that associated endophytes increased the potential fitness of rice plants by enhancing growth, development, biomass and yield in the presence and absence of stress as observed under laboratory and greenhouse conditions (Redman et al., 2011). Interactions between endophytic fungi and their hosts are complex that includes mutualism, commensalism, latent and virulent pathogens (Hallmann et al., 1997; Schulz et al., 1999). From several investigations, there is now growing evidence that the endophytic fungus represents formerly uncharted fungal lineages and comprises vast amounts of fungal diversity in associated plants (O'Brien et al., 2005; Arnold and Lutzoni, 2007; Monnanda et al., 2014). Therefore, there is a high probability of discovering potential endophytic fungi with major application in all sectors including agriculture, therapeutic and for commercial exploitation.

The significant contribution of fungal endophyte in agriculture is its ability to act as a biocontrol agent against a wide range of microbial pathogens, insects, nematodes, and pest. Most importantly, fungal endophyte mediates induced systemic resistance in plants (Arnold and Herre, 2003; Bailey et al., 2006; Nassimi and Taheri, 2017) which is an important mechanism for plant protection and disease management. Yuan et al. (2017), reported endophytic fungi *Penicillium simplicissimum, Leptosphaeria* sp., *Talaromyces flavus*, and *Acremonium* sp. isolated from cotton roots significantly control Verticillium wilt of cotton. There is also a study reporting on a novel endophyte, *Curvularia* sp., that increases host heat tolerance (Redman et al., 2002). *Microdochium bolleyi*, an endophytic fungus of *Fagonia cretica*, displayed antifungal activities against plant pathogen *Microbotryum violaceum* (Zhang et al., 2008). The endophyte *Herbaspirillum seropedicae* and *Clavibacter xylii* have been genetically modified to produce and excrete the δ-endotoxin of *Bacillus thuringensis* to control insect pests (Downing et al., 2000). Hence, all the study possibly established the hidden potential of endophyte and its application in several fields for the benefit of humankind. Saikkonen reported that host-plants without fungal endophyte could not withstand the extreme waves of temperature, drought, salinity and pathogen attack (Saikkonen et al., 2010). Studies on the diversity and isolation of endophytic microbes have been conducted mainly in agricultural and horticultural plants owing to their applied purposes (Hallmann et al., 1997; Sturz et al., 2000). Endophyte research on the development of novel biocontrol agent is still a brooding area that requires much work. Therefore, exploring endophyte from different ecological niches should be encouraged.

Rice (*Oryzae sativa* L.) and maize (*Zea mays* L.) are the two most important cereal crops (Ngachan et al., 2008) with high nutritional value and rice being the staple food of our region has huge demand following maize crop. In this region of North East India, no work on beneficial endophytic fungi and its role in plant health and yield improvement, in particular, are explored. The association of rice with arbuscular mycorrhizal fungi, actinomycetes and endophytic bacteria has been well studied (Glassop et al., 2007; Mano and Morisaki, 2008; Mattos et al., 2008). However, few data are available on rice fungal endophytes especially in the North East region of India. There are study reports on the beneficial contribution of endophytes on overall plant health of rice, endophytic fungi *Phoma glomerata* LWL2 and *Penicillium* sp. LWL3 reporting significant growth promotion of the shoot and associated growth attributes of GAs-deficient dwarf mutant Waito-C and Dongjin-Beyo rice (Muhammad et al., 2012). Also in maize, the study of endophyte is mainly concentrated on root isolates (Orole and Adejumo, 2009; Amin, 2013) with useful aspects of the host plant. The present study focuses mainly on targeting significant endophytes from a local variety of rice and maize grown in the Indian region of Indo-Burma biodiversity hotspot with special reference to Manipur having disease control ability, plant growth promoting potential with a broad host range and better option to promote organic farming. Feng pan in his study pointed out the indispensable role of fungal endophyte in biocontrol and renewed attention being paid to the study focussing on the improved host disease resistance mechanism, alongside the secondary metabolites produce by them being a continuous source of new lead compounds and chemical entities in the fields of agriculture and medicine (Pan et al., 2016). The opportunity to discover new endophytes with promising properties from this unique ecosystem is appealing. Every endophytic microbe contribute to their host defense by certain hidden mechanism yet to be clearly understood and there are many unsolved queries in the endophyte research, therefore our study aims at tapping the endophytic fungal communities associated with different tissue parts of healthy rice and maize crops using morphological and molecular approach for assessing stress tolerance, plant growth promotion, root colonization ability and biocontrol potency against emerging and well-established phytopathogens of rice and maize, *viz*., *Pythium ultimum, Sclerotium oryzae, Rhizoctonia solani*, and *Pyricularia oryzae*. In addition, India being a tropical country with great variation in biodiversity and Manipur, which is listed in the top 10 hotspot biodiversity rich zones of word famous 34 biodiversity hotspots, falling under the Indo-burma region hotspot offer more chances to explore more endophytes that become the solution to many queries of endophytes associated activities.

## Materials and methods

### Climatic condition of the study area

Manipur is located in North Eastern of the Indian subcontinent at Indo-Burma biodiversity hot spot region (elevation of 790 meters) above the sea level with latitude ranging from 23°83′N-25°68′N and longitude 93°03′E–94°78′E, annual rainfall varies from 1,467.5 to 2,593 mm and average weather conditions ranges from −2°C to 34°C. The climate is largely influenced by the topography of the hilly region and ranges from tropical to temperate with rich forest ecosystem. Such variation allows the diverse microbial habitat to adapt various environmental conditions and nature has bestowed the region to study with a good source of genetically and ecologically varied microorganisms.

### Sample collection, endophyte isolation and identification

#### Sample collection and surface sterilization

Healthy indigenous rice variety (Moirangphou) and maize (Chahou chujak) popularly grown in Manipur were sampled during June 2011–October 2012 from various locations having different geographical features. Rice sampling was done during the late reproductive and early ripening phase of growth and for maize, sampling was done during the reproductive phase of growth. The freshly collected samples were brought to the laboratory in the sterile package system and process within 24–48 h of collection.

**Figure 1:**
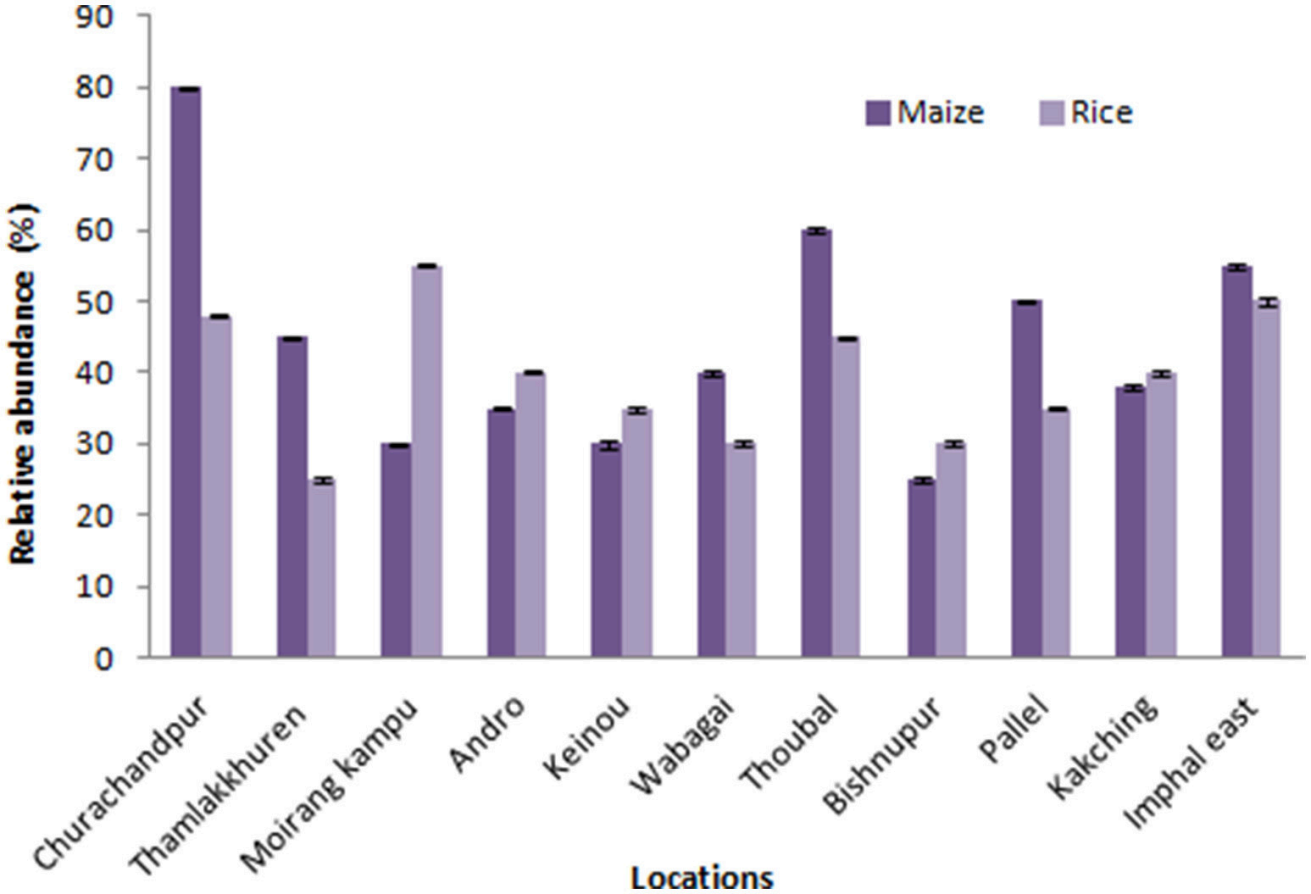
**Relative abundance of endophytic fungal isolates of maize and rice from different locations of Manipur**. The bars represents 11 locations of sample collection sites. Error bar represents Standard error.

Surface sterilization was carried out following modified protocol (Qin et al., 2009; Sarangthem and Momota, 2012). The freshly collected samples were washed thoroughly with the running tap water to remove adhering soil along with associated unwanted particles and soaked for 10 min in distilled water containing a few drops of tween 80. Leaves, stems and roots were cut into appropriate segments and washed twice with sterile distilled water before proceeding for surface sterilization using 80% ethanol for 1 min (leaf), 2 min (stem) and 3 min (root) depending on plant parts. The samples were treated with 4% sodium hypochlorite (Merck, Germany), rinsed with sterile distilled water and treated with 70% alcohol for 1 min, followed by 8–10 successive rinses with sterile distilled water and dried in a sterile condition. The indigenous method followed were (i) direct plate impression of sterilized tissues: The samples were carefully made into thin slice, removing the outer cover and placed on potato dextrose agar (PDA), sabouraud dextrose agar with chloramphenicol (SDA), corn meal agar (CMA), malt extract agar (MEA), czapekdox agar, yeast extract mannitol agar (YEMA) and oat meal agar (OMA). Further, the plates were incubated at 28°C ± 2°C for 2 weeks until the observation of fungal growth. (ii) Spread and pour plate technique: The sterile tissues after aseptically processing and removing the outer edged portion was homogenized using sterile mortar and pestle. The sample extract was directly plated on the media (50 μl) in one set and in another set 1 ml of the sample suspended in 9 ml saline until 10^−5^ dilutions and isolation were done through the spread plate and pour plate techniques. The plates were incubated at 28°C ± 2°C with regular monitoring for 2 weeks or until the observation of fungal growth. Surface sterilized tissues and aliquot from the final rinse was tested as a sterility check measure (Schulz et al., 1998). The success of the surface sterilization method was confirmed by the absence of any microbial growth on the triplicate media plates impregnated with 50 μl aliquots of the final rinse water. The colonization rate of endophytic fungi collected from different location was calculated using the formula given by Petrini et al. (1982) as the total number of representative maize and rice segments colonized by endophyte divided by the total number of segments incubated. Colonization rate was expressed as percentages (Figure 1). All the isolates were deposited in the Microbial Repository Centre, Institute of Bioresources and Sustainable Development (IBSD), Imphal, India.

#### Morphological features studied on different growth media

The endophytic fungal isolates grown on different media were studied for their growth characteristics such as mycelium type, colony color and growth rate on different carbon source. Microscopic identification of the complex spore, hyphae arrangement and reproductive structures were analyzed using Axio vision upright Microscope (Carl Zeiss, Germany).

#### DNA extraction, amplification, and analysis

Genomic DNA was extracted following the modified method adopted from the National Bureau of Agriculturally Important Microbes (NBAIM) U.P, India. Pure fungal cultures were grown on 80 ml potato dextrose broth (PDB) incubated at 30°C ± 2°C incubator shaker revolving at 90 rpm for 7 days. The mycelium was collected by filtration, dried and homogenized properly to avoid shearing of DNA using sterile mortar and pestle. Grind mycelium was dissolved in lysis buffer (TrisHcl-0.788 gm/100 ml, EDTA-1.861 gm/100 ml, SDS-3%, 2-β-mercaptoethanol-1%, Ph -8) and incubated for 1 h at 65°C water bath, equal volume of Tris- Saturated phenol (pH 7.9-8.1) was added and centrifuge at 12,000 rpm for 15 min at 4°C (the centrifuged temperature and time was maintained at 4°C and 15 min respectively throughout the process). The aqueous phase was collected to a fresh tube and an equal volume of phenol: chloroform: isoamylalcohol solution (25:24:1) was added and centrifuge as above. The supernatant was treated with 1/2 volume of chloroform x 1/10 sodium acetate (pH5.2) and Centrifuged at 1,000 rpm. To the supernatant, 500 μl of 70% ice cold alcohol was added and kept at 4°C for 20 min and centrifuged at 12,000 rpm again. The final supernatant was discarded and the pellets were air dried in a sterile condition and dissolved in 50 μl sterile deionised distilled water. DNA samples were further treated for purity and integrity checked by gel electrophoresis before storing at 4°C. The target region of rDNA ITS (ITS1, 5.8S, ITS2) was amplified using primers ITS1 and ITS4 (White et al., 1990). The PCR product, along with the reference marker (100 bp ladder, Promega) was resolved by gel electrophoresis on a 1.5% resolution agarose gel (Tris borate EDTA) for 40 min at 80 V and the amplified bands were visualized under UV light using a Gel imaging system (BioRad, Chemi Doc, MP).

#### Sequence and phylogenetic analysis

The sequences were matched against the nucleotide database using the Basic Local Alignment Search Tool (BLASTn), US National Centre for Biotechnology Information (NCBI), (http://www.ncbi.nlm.nih.gov/BLAST/) for identification of the endophytes. The sequences were aligned using ClustalW-Pairwise Sequence Alignment of the EMBL Nucleotide Sequence Database. The sequence alignments were trimmed and verified by the MUSCLE (UPGMA) algorithm (Edgar, 2004) using MEGA5 software (Tamura et al., 2011). The sequences were considered to be conspecific (Yuan et al., 2010) when the similarity between a particular target sequence and a phylogenetically associated reference sequence are ≥ 99%. The phylogenetic tree was reconstructed and the evolutionary history was inferred using the Neighbor-Joining method (Saitou and Nei, 1987). The robustness of the internal branches was also assessed with 1,000 bootstrap replications (Felsenstein, 1985). The evolutionary distances were computed using the Maximum Composite Likelihood method (Tamura et al., 2004) and were calculated in the units of the number of base substitutions per site. The sequences of this study were deposited in the EMBL-Bank. The accession numbers are detailed in Table 1.

**Table 1:** **Endophytic fungi isolated from apparently healthy tissues of rice and maize plants**.

**Sl no**.	**Culture ID**	**Species identified**	**Tissue part**	**GenBank accession no**.	**Location**	**Max. identity (%)**
1	IBSD-ENF-1	*Gibberella fujikuroi*	Maize root	JX480547	Churachandpur	100
2	IBSD-ENF-2	*Gibberella intermedia*	Maize root	JX480548	Churachandpur	98
3	IBSD-ENF-3	*Fusarium concentricum*	Maize stem	JX480549	Churachandpur	100
4	IBSD-ENF-4	*Aspergillus tubingensis*	Maize root	JX480550	Churachandpur	100
5	IBSD-ENF-5	*Trichoderma koningiopsis*	Maize stem	JX480551	Churachandpur	100
6	IBSD-ENF-6	*Fusarium* sp.	Maize stem	JX480552	Churachandpur	99
7	IBSD-ENF-7	*Fusarium oxysporum*	Maize node	JX480553	Churachandpur	100
8	IBSD-ENF-8	*Fusarium oxysporum*	Maize leaf	JX480554	Churachandpur	100
9	IBSD-ENF-9	*Sarocladium zeae*	Maize leaf	JX480555	Thamlakkhuren	95
10	IBSD-ENF-10	*Fusarium oxysporum*	Maize stem	JX480556	Moirang Kampu	100
11	IBSD-ENF-11	*Fusarium oxysporum*	Maize root	JX480557	Imphal East	100
12	IBSD-ENF-12	*Fusarium oxysporum*	Maize root	JX480558	Imphal East	100
13	IBSD-ENF-13	*Fusarium oxysporum*	Maize root	JX480559	Moirang Kampu	100
14	IBSD-ENF-14	*Fusarium oxysporum*	Maize stem	JX480560	Thoubal	100
15	IBSD-ENF-15	*Fusarium oxysporum*	Maize root	JX480561	Thoubal	100
16	IBSD-ENF-16	*Fusarium oxysporum*	Maize root	JX480562	Thoubal	100
17	IBSD-ENF-17	*Galactomyces geotrichum*	Rice stem	JX480563	Thoubal	100
18	IBSD-ENF-18	*Phoma* sp.	Rice stem	JX480564	Thoubal	99
19	IBSD-ENF-19	*Fusarium sacchari*	Maize leaf	JX480565	Andro	100
20	IBSD-ENF-20	*Fusarium oxysporum*	Maize root	JX480566	Andro	100
21	IBSD-ENF-21	*Talaromyces pinophilus*	Maize root	JX480567	Andro	99
22	IBSD-ENF-22	*Penicillium simplicissimum*	Rice root	JX480568	Keinou	100
23	IBSD-ENF-23	*Fusarium oxysporum*	Rice root	JX480569	Keinou	100
24	IBSD-ENF-24	*Gibberella moniliformis*	Maize leaf	JX480570	Thoubal	100
25	IBSD-ENF-25	*Fusarium oxysporum*	Rice stem	JX480571	Keinou	100
26	IBSD-ENF-26	*Fusarium* sp.	Maize root	JX480572	Moirang Kampu	100
27	IBSD-ENF-27	*Fusarium oxysporum*	Maize leaf	JX480573	Imphal East	100
28	IBSD-ENF-28	*Epicoccum sorghi*	Maize root	JX480574	Kakching	95
29	IBSD-ENF-29	*Fusarium denticulatum*	Maize root	JX480575	Thamlakkhuren	99
30	IBSD-ENF-30	*Gibberella intermedia*	Maize root	JX480576	Imphal East	99
31	IBSD-ENF-31	*Acremonium* sp.	Maize leaf	JX480577	Wabagai	100
32	IBSD-ENF-32	*Eupenicillium javanicum*	Maize leaf	JX480578	Thamlakkhuren	100
33	IBSD-ENF-33	*Fusarium andiyazi*	Maize stem	JX480579	Imphal East	100
34	IBSD-ENF-34	*Fusarium incarnatum*	Maize root	JX480580	Imphal East	100
35	IBSD-ENF-35	*Fusarium equiseti*	Maize leaf	JX480581	Imphal East	100
36	IBSD-ENF-36	*Aspergillus carneus*	Maize stem	JX480582	Wabagai	99
37	IBSD-ENF-37	*Fusarium oxysporum*	Maize stem	JX480583	Wabagai	100
38	IBSD-ENF-38	*Gibberella moniliformis*	Maize root	JX480584	Bishnupur	100
39	IBSD-ENF-39	*Sordariomycetes* sp.	Maize leaf	JX480585	Pallel	99
40	IBSD-ENF-40	*Eutypella scoparia*	Maize leaf	JX480586	Wabagai	100
41	IBSD-ENF-41	*Sarocladium strictum*	Maize node	JX480587	Imphal east	97
42	IBSD-ENF-42	*Penicillium ochrochloron*	Maize root	JX480588	Imphal east	99
43	IBSD-ENF-43	*Gibberella intermedia*	Maize root	JX480589	Imphal east	100
44	IBSD-ENF-44	*Rhizomucor* sp.	Maize leaf	JX480590	Imphal east	87
45	IBSD-ENF-45	*Sarocladium zeae*	Maize stem	JX480591	Imphal east	96
46	IBSD-ENF-46	*Gibberella intermedia*	Maize root	JX480592	Imphal east	100
47	IBSD-ENF-47	*Fusarium* sp.	Maize root	JX480593	Imphal east	100
48	IBSD-ENF-48	*Fusarium* sp.	Maize stem	JX480594	Imphal east	99
49	IBSD-ENF-49	*Aspergillus ustus*	Rice leaf	JX480595	Thamlakkhuren	99
50	IBSD-ENF-50	*Fusarium succisae*	Maize stem	JX480596	Imphal east	100
51	IBSD-ENF-51	*Gibberella circinata*	Maize stem	JX480597	Bishnupur	100
52	IBSD-ENF-52	*Sarocladiumzeae*	Maize leaf	JX480598	Bishnupur	100
53	IBSD-ENF-53	*Fusarium oxysporum*	Maize leaf	JX480599	Pallel	99
54	IBSD-ENF-54	*Pleosporales* sp.	Maize leaf	JX480600	Kakching	99
55	IBSD-ENF-55	*Penicillium* sp.	Maize leaf	JX480601	Kakching	99

### Stress tolerance, biocontrol, and growth promotion assays

#### Stress tolerance activity

All the isolates were subjected to abiotic stress tolerance study against a different range of pH, salt and temperature. The test cultures were exposed to different growth conditions to salt stress, ranging from 3 to 10% sodium chloride incorporated Potato dextrose agar. The test endophytes were spot inoculated on the plates and incubated for 2 weeks and the growth was recorded. For stress tolerance study on pH (2 to 12), Potato Dextrose Broth was adjusted using glacial acetic acid (Sigma-Aldrich, USA). Test cultures were inoculated and incubated for 2 weeks and growth was monitored through optical density. Temperature stress tolerance was studied at 4°C, 8°C, 10°C, 45°C, and 50°C. Potato Dextrose Agar plates inoculated with test organisms were incubated at the respective temperatures for the observance of growth. The overall growth condition of the endophytes was observed and assessed based on their ability to grow in such extreme conditions.

#### *In vitro* antagonism assay

The endophytes were evaluated for their antagonistic activity against widely prevailing pathogens of cereal crops *viz. Pyricularia oryzae* (ITCC No. 4511), *Rhizoctonia solani* (ITCC No. 6491), *Sclerotium oryzae* (ITCC No. 4107) and *Pythium ultimum* (ITCC No. 1650) obtained from Indian Type Culture Collection (ITCC), New Delhi, India. Antagonistic activity of the endophytes was checked using a dual culture plate assay (Coskuntuna and Ozer, 2008). Fungal discs (5 mm) of the pathogen and test organism were inoculated at opposite sides of PDA plates with the partition gap of 3 cm approximately and incubated at 28°C ± 2°C. Plates inoculated only with the pathogen served as control. The inhibition percentage was calculated using the formula given by (Fokkema, 1976), Inhibition % = C − T/Cx100, here, “C” represents the growth diameter of the pathogen in the control plate and “*T*” represents the pathogen diameter growth on the dual plate, where both the test endophyte and the pathogen were inoculated.

#### The qualitative IAA production assay

The IAA production assay was done following the protocol of Bric et al. (1991). Luria Bertani broth (LB), LB incorporated with 5 mM L-tryptophan (LBT) and LBT incorporated with 0.05% sodium dodecyl sulfate and 1% glycerol was used for screening potential fungal isolates for qualitative IAA production. Cultures were inoculated on the media overlaid with an 82-mm-diameter disk of sterile nitrocellulose membrane and incubated inversely at 28 ± 2°C until the desired growth was observed. The membrane disc was removed from the plate and treated with Salkowski reagent. IAA producing cultures form a characteristic red halo zone within the membrane immediately surrounding the colony. The percentage of IAA was measured by subtracting the culture colony diameter against the halo diameter (HD).

#### Growth assay on nitrogen free media

Burk's nitrogen free media (Merfat, 2008) and Norris glucose nitrogen free media (HiMedia, India) were used to assay the growth of the isolates with or without the addition of ammonium chloride, as a unique nitrogen source (Dobereiner, 1995). The observation was made after 7 days of incubation at 30°C ± 2°C based on the type of growth morphology and appearance as a qualitative study on nitrogen fixation.

### Disease suppressive mechanisms

#### Phosphate solubilization and protease production assay

The modified Pikovskaya agar was used, with the addition of 0.3% insoluble calcium triphosphate (HiMedia, India). The test cultures were inoculated and incubated at 28°C ± 2°C until growth appears. The presence of halo zone or clearance around the colony after 7 days incubation qualitatively ensures phosphate solubilizing potential. Pure fungal endophytic isolates were inoculated on Skim milk agar (HiMedia, India) following modified protocol by Rodr'guez and Fraga (1999) and incubated at 28°C ± 2°C until the colony was observed. Visible clearance around the colony has qualitatively indicated protease activity. The P solubilization and enzyme production were determined by subtracting the diameter of the fungal colony from the diameter of the total zone.

#### Chitinase production assay

Chitin detection media were prepared with slight modification from the method given by Agrawal and Kotasthane (2009). Colloidal chitin and indicator dye bromocresol purple was incorporated in the media for studying chitin utilization. Plates were inoculated with pure fungal endophytes and incubated at 28°C ± 2°C for 5 days. The presence of color change from yellow to purple color around the colony indicates positive chitinase activity.

#### Production of β-1, 3-glucanase

Carboxymethylcellulose agar incorporated with laminarin (Sigma) was used for β-1, 3-glucanase detection assay according to the modified method of Katatny et al. (2001). The plates were incubated at 28°C ± 2°C for 4–5 days. The colony was flooded with a 0.1% congo red dye for 15 min and washed with 1N NaCl and 1N NaOH for 15 min respectively till the appearance of a clear zone around the colony. Clear zone indicates positive activity. The zone diameter was determined by subtracting the diameter of the fungal colony from the diameter of the total clear zone.

#### Siderophore production assay

The fungal isolates were inoculated on Chrome azurol S (CAS) agar medium and incubated at 28°C ± 2°C for 7 days. The assay is based on the competition for iron between the ferric complex of the indicator dye, CAS and a chelator or siderophore produced by the microorganisms. The chrome azurol S agar was prepared following the modified protocol of Schwyn and Neilands (1987) in which 60.5 mg CAS was dissolved in 50 ml distilled water and mixed with 10 ml iron (III) solution (1 mM FeCl_3_.6H_2_O, 10 mM HCL). With constant stirring, the solution was slowly added to 72.9 mg of hexadecyltrimethylammonium bromide (HDTMA) indicator dissolved in 40 ml of water and autoclaved at 121°C for 15 min. The basal media were prepared using succinic acid 0.5%, K_2_HPO_4_ 0.4%, (NH_4_)_2_SO_4_.7H_2_O, agar 2% at pH 5.3±2. The partially cooled autoclaved blue agar was added to the basal media and slowly mixed until it gives the desired blue color agar and finally poured onto the plates. The colonies turning yellow color were considered siderophore producing. The CAS reaction rate was determined by the color change from blue to yellowish orange, purple or dark purplish red and for non-siderophore producing organisms no color change was observed. The zone diameter was determined by subtracting the diameter of the fungal colony from the diameter of the total color zone.

#### Production of cellulase and amylase enzyme

Aneja (2003) screened the fungal isolates for the production of cellulase enzyme using carboxymethyl cellulose (CMC) following a modified protocol. Minimal synthetic chemicals comprising of 0.2% NaNO_3_, 0.8% K_2_HPO_4_, 0.1%Mg.SO_4_.7H_2_O and 0.8% KCl with the addition of peptone 0.2%, glucose 0.1%, and 0.5% CMC. K_2_HPO_4_ and CMC solution were prepared separately; K_2_HPO_4_ solution was dissolved in the composition followed by CMC solution by stirring continuously, final pH was adjusted to 5.3 ± 2. Isolates were inoculated on the CMC plates and incubated at 28°C ± 2°C until the observation of growth. Glucose yeast peptone agar comprising of 1% glucose, 0.2% yeast extract, 0.5% peptone with the addition of 2.5% soluble starch was used to determined amylase activity (Hankin and Anagnostakis, 1975). The plates were inoculated with the cultures and incubated at 28°C ± 2°C until the observance of growth. Both the CMC and amylase plates were flooded with 1% iodine in 0.5% potassium iodide solution for a few seconds and drained off to observe a clear halo zone around the colony determining the positive enzyme production. The zone diameter was determined by subtracting the diameter of the fungal colony from the diameter of the total halo zone.

#### Test for HCN production

The hydrogen cyanide production test was performed using a modified protocol of Miller and Higgins (1970). HCN medium was prepared using 0.3% Picric acid solution along with 1.5% sodium carbonate. To the solution, sterilized strips of Whatman filter paper No. 1 (China) were soaked and dried in a sterile environment. Fungal cultures were inoculated on PDA slant and the treated filter paper strips were placed on the slant simultaneously closing the lid tightly by wrapping with parafilm. The slants were incubated for 7–14 days. The rate of HCN production was determined by the color changes in the filter paper strips, from the original yellow color to brown or reddish brown. Scoring was done as weak (yellow to light red), moderate (brown), and strong (reddish brown).

### Root colonization assay

Qualitative assessment of root colonization ability by the isolates was performed following modified protocol by Landa et al. (2002). Based on the multiple enzyme assays and antagonistic activity, ENF31 (E2), originally isolated from maize and ENF22 (E1), isolated from rice was selected for root colonization assay in *in situ* condition on maize and rice plants. The antibiotic mutated cultures were grown in potato dextrose broth (PDB^+++^) supplemented with ampicillin (100 μg/ml), chloramphenicol (30 μg/ml), and rifampicin (100 μg/ml) in shaking incubator at 140 rpm for 72 h. The cell pellets were collected and washed twice in sterile water by centrifugation at 7,000 × g for 8 min and resuspended in sterile distilled water. Cell densities were adjusted to 10^6^ fungal cells per ml mixed with 1% carboxymethylcellulose suspension (50 ml of suspension per 500 g of soil) to give approximately 10^6^ CFU/ml fresh weight of soil. Experimental pots filled with 300 g of treated sterilized soil were shown with 6 sterilized seeds in each set of the pot. The root colonization ability was studied with test endophytes (E1, E2) and without tests endophytes as a control (C), endophytes with *Rhizoctonia solani* (E1+RS; E2+RS), and endophytes with *Sclerotium oryzae* (E1+SO; E2+SO). The experiment was conducted in triplicates. The experiment was carried out in a growth chamber at 25–30°C with a 12 h photoperiod. Pots were covered with sterile plastic until the emergence of the seedling. Plants were allowed to grow for 3 weeks and watered with sterile distilled water once in 2 days. Two plants were selected randomly from each pot at the end of the 3-week cycle to determine the population size of the introduced fungal endophytes. The initial growth response after the colonization was observed. The root portion with adhering rhizosphere soil was collected and dispensed in 50 ml falcon tube containing 10 ml sterile distilled water. The inoculum recovery was carried out on the 7th and 30th day old samples (**Table 3**). The sample roots were collected, surface-sterilized, macerated and plated in the concerned media, also, the rhizosphere soil adhering roots were carefully vortex and sonicated for 1 min and the wash solution was then processed for checking inoculum recovery by the following methods (i) 1 ml of the washed solution was serially diluted with 9 ml sterile distilled water till 10^−5^. (ii) 1 ml of the washed solution was serially diluted to 9 ml of PDB^+++^ broth till 10^−5^. 100 μl of the sample was plated in duplicates on antibiotic free PDA and PDA^+++^ agar and incubated at 28 ± 2°C.

### Evaluation of fungal diversity

The diversity of endophytic microorganisms associated with different tissue sections of rice and maize was evaluated using Shannon-Weiner Diversity Index (*H*′) that takes into account both species richness and evenness (Tao et al., 2008).

### Statistical analysis

All the test assays were performed in triplicates. Descriptive statistics were used to study the rate of inhibition against the tested phytopathogens and to determine the enzyme assays (means and standard error) of the endophytic isolates. The mean comparison was performed by Fisher's protected least significant difference (LSD) test at *P* = 0.05.

## Results

### Isolation and diversity analysis

One Hundred and twenty-three different fungal species were isolated from 930 tissue sections of indigenous rice and maize plants collected from various locations of Manipur (Figure 1). Endophytic fungal occurrence in maize was highest from Churachandpur (25), followed by Thoubal (21) wherein for rice, Moirangkangpu (23) yields highest fungal isolates followed by Imphal East (21). Values on analysis of Shannon-Weiner Diversity Index (*H*′), of maize (2.34, 0.099, 0.900) and rice (2.37, 0.093, 0.906) indicates that the prevalence of endophyte diversity is more with rice than maize crops. The diversity analysis study of maize, values of *H*′, for roots (1.38, 0.25, 0.74), stems (1.37, 0.21, 0.78) and leaves (1.57, 0.15, 0.84) reveals leaves tissues harboring diverse endophytes and equally distributed in roots and stem parts of maize. Study on rice associates reveals diversity is prevalent in rice roots (1.56, 0.18, 0.81) and equally distributed in case of stem (1.331, 0.27, 0.72) and leaves (1.332, 0.21, 0.78). Shannon-Weiner diversity Index on maize supports the highest diversity occurrence of endophytes on leaf section and in the case of rice, root tissue harbors more diverse endophyte and equally distributed in leaf and stem parts. Here, the occurrence of genera *Fusarium, Aspergillus, Penicillium*, and *Acremonium* in the majority of the tissue sections in both the sampled crops also reveal that the recovered fungal endophytes are not tissue and even host specific. This assures wide applicable nature of the isolates on multiple host plant.

### Molecular identification and phylogeny

The amplified product of the rDNA regions (ITS1, 5.8S and ITS2) was sequenced for species identification. The sequences were searched for homology match using the Basic Local Alignment Search Tool (BLASTn) of the National Centre for Biotechnology Information and the endophytes were considered conspecific at a threshold identity of ≥99% when compared to the most closely related strains (Yuan et al., 2010). The sequences have been submitted to NCBI GenBank and the details of the isolates along with the source of collection, tissue origin and accession numbers were highlighted in Table 1. The present result was obtained through culture- dependent approach, with the criteria to assess the overall beneficial quality and to determine the broad host specificity of the fungal endophytic isolates. 55 endophytic isolates (including an outgroup) were identified based on morphology, microscopy and rDNA ITS sequence analysis. Endophytic fungus belonging to the family *Trichocomaceae, Hypocreaseae, Nectriaceae* and *Mucoraceae* were reported for the first time in the cereal crops. *Fusarium, Penicillium, Aspergillus, Acremonium, Trichoderma*, and *Phoma* sp. were among the common genus present. The blast search of the ITS rDNA gene sequence similarity match ranges from 87 to 100% with the probability of new species recovery (Table 1).

**Figure 2:**
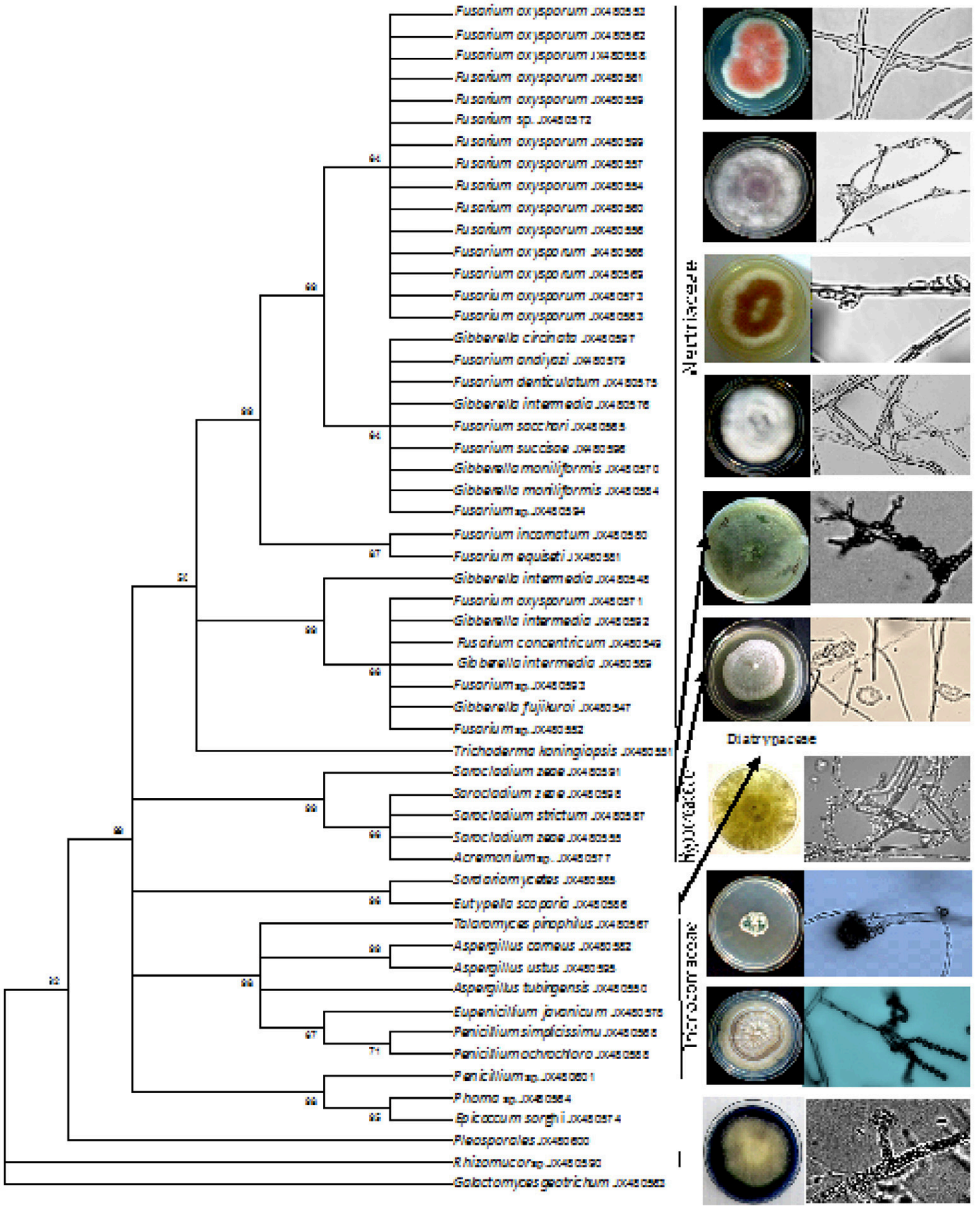
**UPGMA method phylogenetic tree based on rDNA ITS sequences of fungal endophytic isolates obtained from tissue sections of maize and rice**. The percentage of replicate in which the associated taxa clustered together in the bootstrap test (1,000) is shown next to the branches.

The phylogenetic tree was constructed for all the isolates using the UPGMA method (Figure 2). The tree revealed the association and relatedness among the isolates common to both maize and rice samples. A genus of *Fusarium, Penicillium*, and *Aspergillus* were commonly isolated in both the crops whereas *Acremonium* sp. was a frequently isolated from maize. *Phoma* sp. was isolated from rice alone and *Epicoccom sorghi* from maize plant. The evolutionary distances were computed using the Maximum Composite Likelihood Method (Tamura et al., 2004) and represented in the units of the number of base substitutions per site. The percentage of replicated trees in which the associated taxa clustered together in the bootstrap test (1,000 replicates) was shown next to the branches (Felsenstein, 1985) (Figure 2). The analysis involved 55 nucleotide sequences. Evolutionary profile study was conducted in MEGA5 (Tamura et al., 2011).

### Growth promotion, stress tolerance, and disease suppression

The potentials for plant growth promotion and biochemical enzyme activity of the selected fungal endophytes were evaluated. The qualitative IAA production assay was performed for the screening of IAA-producing isolates, IAA production was reported in isolates, ENF 3, ENF5 and ENF22. Among the isolates, ENF5, ENF22, ENF31, and ENF41 grow more abundantly on Burk's nitrogen free media and Norris glucose nitrogen free media and the rest of the fungal endophyte grows in moderate condition. The observation of fungal growth on different concentrations of salt, pH and temperature reveals the tolerance of the isolates on different abiotic stress conditions. Highest growth tolerance was exhibited by ENF 22 and ENF 31 followed by ENF32 under salt stress condition (Table 2). The growth on the temperature at 4°C was negligible and at 10°C, the growth rate was slow. The Maximum growth rate was observed between 20 to 30°C and at 40°C the isolates display moderate rate of growth and the high temperature withstanding capacity at 50°C was observed for fungus- ENF13, ENF22, ENF 31, ENF36, EN44 and ENF49. Endophytes ENF22 (*Penicillium simplicissimum*), ENF31 (*Acremonium* sp.), ENF41 (*Saracladium strictum*), ENF49 (*Aspergillus ustus*) and ENF 53 (*Fusarium oxysporum*) shows the production of all the tested enzymes. Among the isolates, ENF22, ENF31 and ENF52 positively utilized the incorporated phosphate while protease activity was shown for ENF22, ENF31, ENF36 and ENF52, ENF31 showed the highest activity for β-1, 3-glucanase and cellulase activity (Table 2). Maximum chitinase activity was produced by ENF 41, whereas, siderophore and amylase activity was strongest in ENF22.

**Table 2:** **Hydrolytic enzyme activitity and stress tolerance of endophytic fungal isolates**.

**Isolates**	**Qualitative biochemical assays**								**Growth on nfb**	**Stress tolerance-pH. Salt, temperature**								
	**P- solubiliza-tion activity**	**Chitinase**	**Siderophore**	**Protease**	**Beta-1,3 glucanase**	**Cellulase**	**Amylase**	**HCN Produc-tion**		**pH-2**	**pH-3**	**pH-12**	**Nacl 3%**	**Nacl 8%**	**Nacl 10%**	**4°C**	**10°C**	**50°C**
ENF-13	−	3.32 ± 0.014	2.86 ± 0.031	−	−	−	1.14 ± 0.01	−	+	−	++	++	++	+	−	-	+	+
ENF-27	−	3.35 ± 0.027	2.97 ± 0.008	−	−	−	−	−	+	−	+++	+	+++	+	−	−	+	−
ENF-34	−	1.22 ± 0.018	1.23 ± 0.066	−	3.51 ± 0.01	1.16 ± 0.02	3.35 ± 0.027	+	+	−	+++	+++	+++	−	−	−	+	−
ENF-22	1.24 ± 0.02	1.15 ± 0.02	1.88 ± 0.057	1.16 ± 0.026	3.12 ± 0.01	1.70 ± 0.01	2.93 ± 0.031	−	+	++	++	++	+++	++	−	±	+	+
ENF-44	1.33 ± 0.015	2.01 ± 0.06	1.44 ± 0.01	−	1.73 ± 0.065	2.56 ± 0.066	−	−	+	−	+++	+++	++	++	−	−	+	+
ENF-53	2.13 ± 0.02	1.12 ± 0.023	1.17 ± 0.089	1.13 ± 0.003	1.32 ± 0.014	1.11 ± 0.6	1.82 ± 0.012	−	+	−	+++	−	+	+	−	−	+	−
ENF-16	−	3.12 ± 0.029	2.31 ± 0.026	−	4.19 ± 0.012	1.12 ± 0.057	1.89 ± 0.88	+	+	−	+++	++	++	+	−	−	−	−
ENF-32	1.86 ± 0.07	2.2 ± 0.01	−	1.12 ± 0.02	1.85 ± 0.076	1.66 ± 0.1	2.53 ± 0.02	−	+	−	+++	+++	+++	++	−	−	+	−
ENF-33	−	2.45 ± 0.06	2.13 ± 0.014	−	−	2.6 ± 0.057	1.11 ± 0.026	−	+	−	+++	−	+++	−	+	−	−	−
ENF-36	−	1.20 ± 0.014	1.24 ± 0.088	1.10 ± 0.01	4.12 ± 0.063	3.18 ± 0.076	4.04 ± 0.07	−	+	−	+++	+++	++	++	−	−	+	+
ENF-45	−	1.11 ± 0.055	1.42 ± 0.02	1.48 ± 0.6	1.55 ± 0.028	3.12 ± 0.003	3.52 ± 0.026	−	+	−	+++	+++	++	−	−	−	+	−
ENF-49	1.62 ± 0.012	1.13 ± 0.089	0.94 ± 0.003	1.19 ± 0.017	1.17 ± 0.026	1.13 ± 0.046	3.43 ± 0.011	−	+	+	+++	++	++	+	−	−	+	+
ENF-41	1.55 ± 0.031	3.55 ± 0.065	1.22 ± 0.046	2.56 ± 0.065	2.38 ± 0.02	3.14 ± 0.01	1.72 ± 0.011	−	++	+	+++	+++	+++	+	−	+/−	+	−
ENF-31	1.89 ± 0.023	3.35 ± 0.014	1.65 ± 0.008	3.66 ± 0.01	4.36 ± 0.03	3.95 ± 0.029	2.28 ± 0.014	−	+	−	++	++	++	+	+	−	−	−
ENF-5	−	1.76 ± 0.076	1.10 ± 0.028	1.11 ± 0.076	2.26 ± 0.01	3.28 ± 0.6	1.19 ± 0.012	−	++	++	++	++	++	++	−	−	+	+

Result: Significant difference among biochemical assays of the isolates (one−way ANOVA followed by Fisher's protected least significant difference (LSD) test, P < 0.05). Growth on nfb (Nitrogen free basal media) and stress tolerance (−, no activity; ±, tinge growth+, moderate activity;++, high activity;+++, very high activity)

### Evaluation of antagonistic activity and root colonization ability

**Figure 3:**
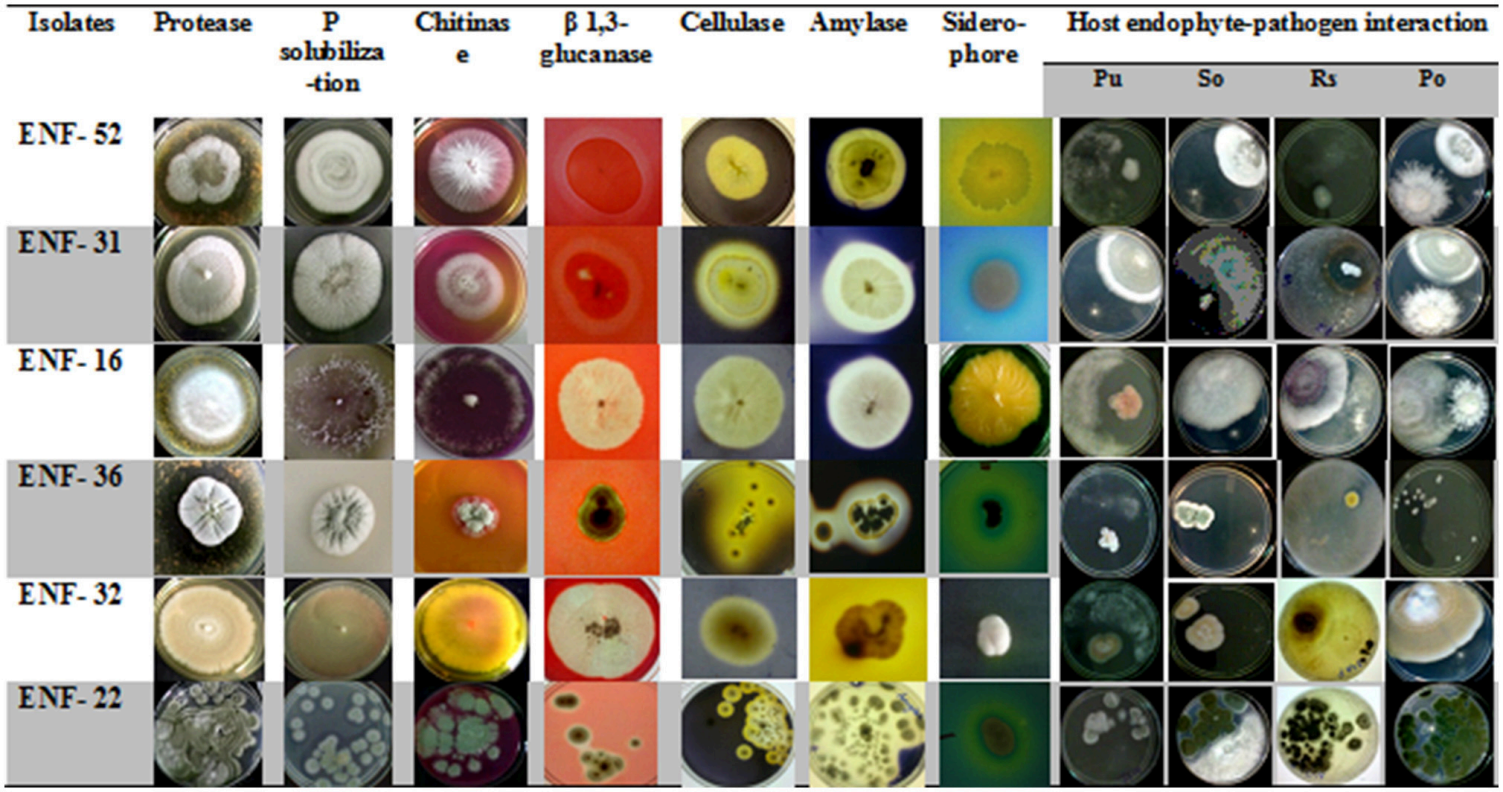
**Plates showing properties of enzyme assays and antagonism activity of the fungal endophytic isolates**. Antagonism toward: PU, *Pythium ultimum*; SO, *Sclerotium oryzae*; RS, *Rhizoctonia solani*; PO, *Pyricularia oryzae*.

**Figure 4:**
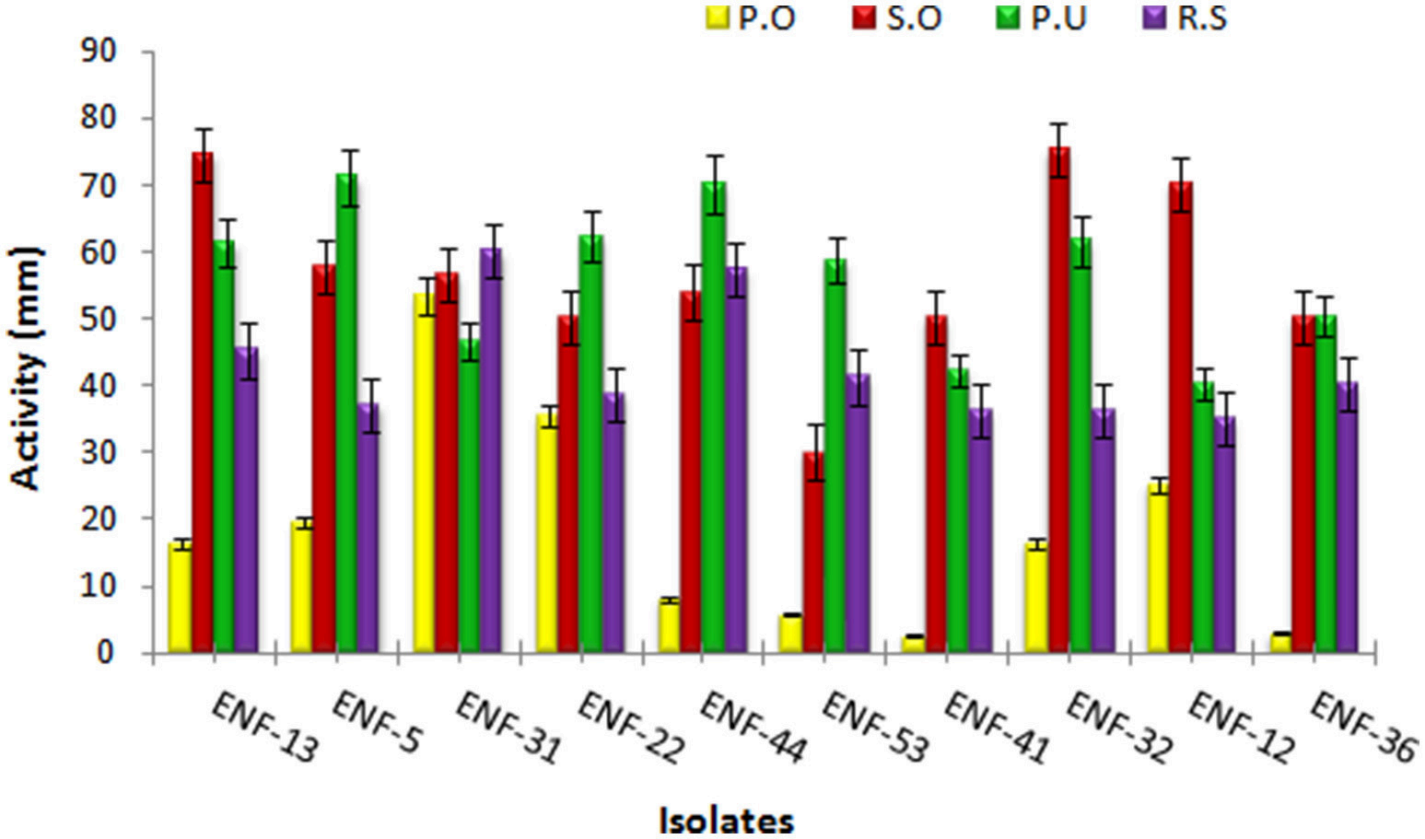
**Antagonistic activity of the endophytic fungal isolates**. The bars represent endophytic fungal isolates. Color indicates the phytopathogens: yellow - *Pyricularia oryzae*, red- *Sclerotium oryzae*, green- *Pythium ultimum and* purple- *Rhizoctonia solani*. Error bar represents Standard error.

Different interaction pattern between the pathogen and the endophyte were observed and analyzed. The zone of inhibition exhibited by ENF22, ENF31, and ENF52 was shown in (Figure 3). The percent antagonism or the growth inhibition percentage is summarized in Figure 4. Observation of statistical data reveals effective control of *Pythium ultimum, Sclerotium oryzae* followed by *Rhizoctonia solani* by all the tested endophytes and poor control was observed for *Pyricularia oryzae*. Among them, ENF22 and ENF31 equally controls all pathogens.

The ability of ENF22 and ENF31 to colonize maize and rice was evaluated over 3 weeks of plant growth. Both the isolates were able to efficiently colonize the respective host plants throughout the phase of the study without any visible disease symptoms and irrespective of the interchange host plants proving its non-host specificity. The isolate ENF22 and ENF31 are at par with significant colonizing ability in the presence of pathogens was shown by ENF22 (Table 3). There is an observation of steady maintenance of the inoculum population throughout the study period. The recovered fungal endophytes were plated on PDB^+++^ plates and microscopically observed for its morphology identification defining the structures of *Penicillium simplicissimum* (ENF22) and *Acremonium* sp. (ENF 31).

**Table 3:** **Endophytic fungal colonies isolated from the roots of rice and maize plants**.

**Innoculum**	**Rice**		**Maize**	
	**7 days**	**30 days**	**7 days**	**30 days**
E1	9.66 ± 0.88	17.66 ± 0.88	10.33 ± 0.88	17.66 ± 1.20
E2	10.66 ± 0.88	16.33 ± 0.88	9 ± 1.15	15.33 ± 0.88
E1+RS	10.66 ± 1.20	17 ± 0.57	9.66 ± 1.45	14 ± 1.15
E1+SO	9.66 ± 0.88	15 ± 1.52	12 ± 0.577	13.66 ± 1.20
E2+RS	7.66 ± 1.20	13.33 ± 1.45	8.33 ± 0.88	14.66 ± 2.02
E2+SO	8 ± 1.15	12.33 ± 1.20	10 ± 1.73	15.66 ± 1.76

*The data represent mean values and standard error of three replicas per treatment, the values in CFUg^−1^ sterilize plant root against initial inoculum of 10^6^ CFU/ml*.

^*^E1, Penicillium simplicisssum; E2, Acremonium sp.; RS, Rhizoctonia solani; So, Sclerotium oryzae.

## Discussion

The world has risen higher with the advancement in technologies, but, the reflection on food productivity with ever increasing demand and the frequent food crisis scenario around the globe is tormenting. In 2012, the United Nation's Food and Agriculture Organization (FAO) estimated that some 920 million people-1/8th of the world's population do not have enough food to meet their daily intake. Global maize, wheat, and rice reserves have hit low production in recent years (Tom and Wolf, 2012). Moreover, the recent turmoil, changes in the overall climatic conditions worldwide have broad a major concern for the survival of plant crops and a running issue of productivity and yield to feed the growing demand. Indeed, researchers are focusing on novel microorganism as a means to better plant health and higher productivity of food crops.

**Figure 5:**
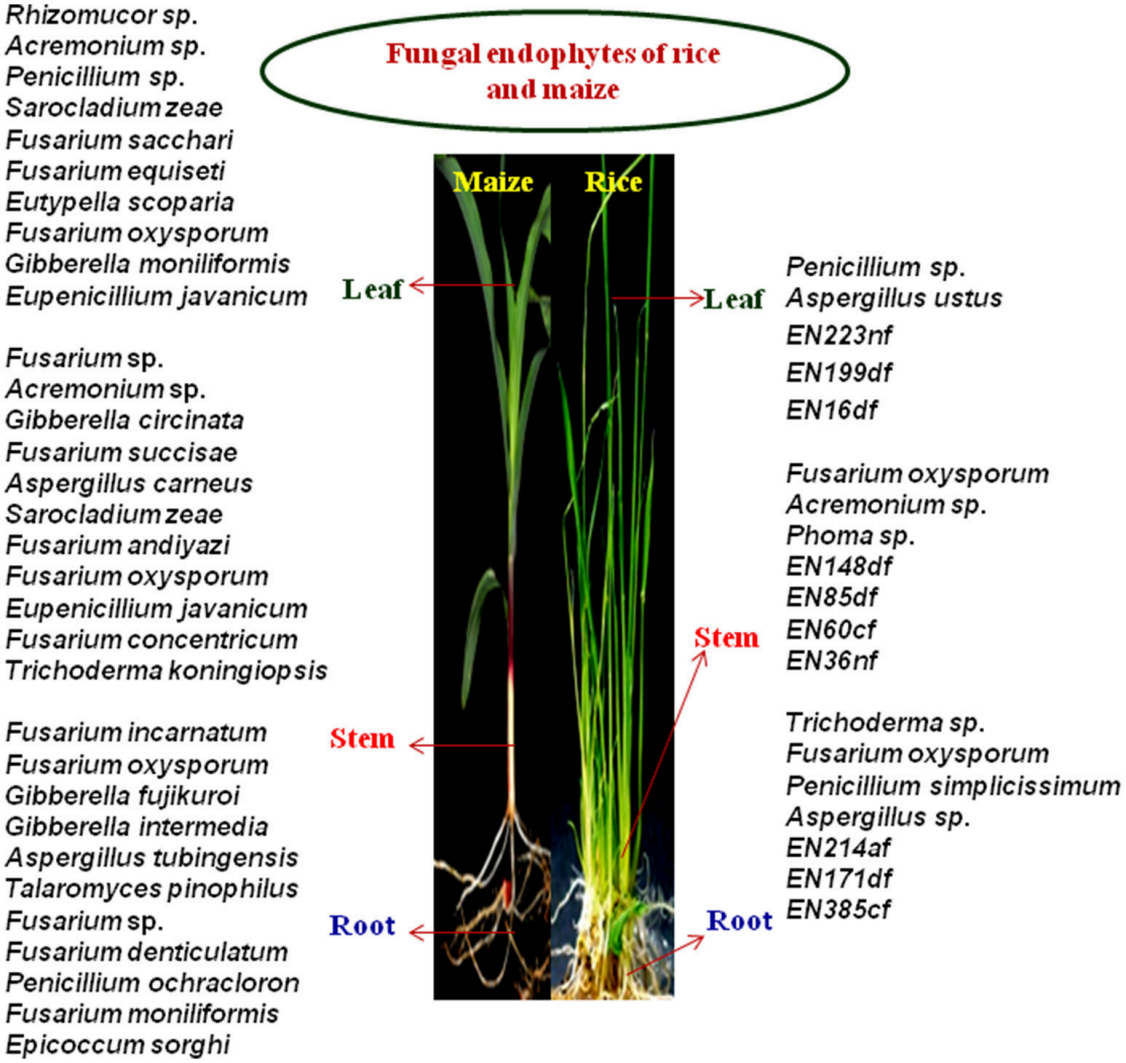
**Endophytic fungal assemblages of Maize and Rice**.

A total of 123 endophytic fungi belonging to different species were isolated from 930 tissue sections of root, leaf and stem of healthy rice and maize samples, out of which, 66 were maize associated and 57 isolates were from rice plants. 55 isolates were screened down for further studies based on *in vivo* biochemical enzyme assays and results on biocontrol activities. In our present study, endophytes belonging to phylum Ascomycota (99%) were found to be the most dominating and least dominance was observed in case of phylum Zygomycota (1%) with just a percent of the total isolate, whereas fungus belonging to class Sordariomycetes, Eurotiomycetes and Dothideomycetes were equally distributed throughout maize and rice plants. Diversity analysis reveals rice harbor more species diversity than maize and in both the crops, Fusarium sp. was frequently isolated. A study conducted by Le, reported that, of the 33 fungal endophytic isolates of rice, *Fusarium* species majorly dominates, followed by few *Trichoderma* sp. (Le, 2006), coinciding with our findings (Figure 5). It has been reported that genus *Fusarium, Trichoderma, Acremonium, Aspergillus, Penicillium, Botryodiplodia, Alternaria alternata, Phoma* sp. and *Beaveria bassiana* were endophytes isolated from maize roots (Orole and Adejumo, 2009; Amin, 2013) also, *Trichoderma* sp., *Fusarium* sp., *Acremonium* sp., and *Aspergillus* sp. were isolated from cacao fruit and leaves, whereby Acremonium sp. is reported as a potential biological control agent against cocoa pod borer, *C. cramerella* (Amin, 2016), where most of them are related to our existing isolates. A genus belonging to *Acremonium, Fusarium* and *Penicillium* were found most dominated in maize leaf. Common endophyte isolated from tissue sections of rice and maize showed the presence of *Aspergillus, Penicillium* and *Fusarium* sp. as a root-associated endophyte. *Fusarium* sp. and *Acremonium* sp. were found to be more dominating in stem followed by *Penicillium* and *Aspergillus* in rice leaf. A study conducted in China on fungal endophytes isolated from healthy paddy plants reported genera *Aspergillus* and *Penicillium* were also among the most common endophytes besides *Fusarium* (Tian et al., 2004). Less common associated endophytes are *Trichoderma* sp., *Eutypella scoparia, Phoma* sp., *Epicoccum sorghi* and *Rhizomucor* sp. (ENF44). Interestingly, ENF44, showed 87% identity matched to the reference strain, *Rhizomucor* sp. Rhi25 (JQ582428.1) of NCBI and hence, the possibility of a new species is predictable. The presence of *Fusarium* sp., *Penicillium* sp., *Acremonium* sp. and *Aspergillus* sp. throughout the plant parts showed its close association with the cereal host. The general recovery of *Fusarium* sp. from different plant as broad host range endophyte (Shahasi et al., 2006; Changhong et al., 2010) was reported by many authors and there were numbers of research articles reporting *Fusarium* sp. as beneficial biocontrol agent and a source of bioactive molecules (Shweta et al., 2010; Tayung et al., 2011). The biodiversity analysis using Shannon-Weiner Diversity Index confirms that rice samples harbor a higher diversity than maize. To our knowledge, this is the first report carried out from this region on bioprospecting of endophytic fungal microbes of maize and rice for agricultural application.

Another factor that encourages endophyte study is the importance of the organic approach in agricultural food crops and combat the use of chemicals that have anti-ecological consequences that directly or indirectly impact the environment and ultimately human health. Therefore, the used of microbial resources as biocontrol agents for controlling diseases, growth promotion and increased yield are an alternative need. The present study employs four phytopathogens with unique characteristics widely prevalent worldwide among the cereal crops, *viz*., *Rhizoctonia solani, Pyricularia oryzae, Pythium ultimum*, and *Sclerotium oryzae*. The tendency of growth pattern and interaction toward the test pathogen and host-endophyte was evaluated macroscopically and microscopically referring to the endophyte-pathogen interaction studies given by Miles et al. (2012). ENF5 (*Trichoderma koningiopsis*), ENF16 (*Fusarium oxysporum*), ENF22 (*Penicillium simplicissimum*), ENF32 (*Eupenicillium javanicum*), ENF31 (*Acremonium* sp.) and ENF33 (*Fusarium andiyazi*) were among the dominant antagonists. A study reported, production of pyrrocidines A and B, by *Acremonium zeae*, which augments host defense against microbial pathogens causing seedling blights and stalk rots acting as a protective endophyte of maize (Wicklow and Poling, 2009). Rice isolate, *Penicillium simplicissimum*, could tolerate salt up to 10%, the important factor for further study, the salt tolerating capacity of *Penicillium* sp. is also reported in a study performed by Khan et al. (2012), stating that endophytic fungal symbiosis of *Penicillium minioluteum* under abiotic salinity stress condition could increase the Daidzein and Genistein contents in the soybean when compared with control plants and thus rescued soybean plant growth by influencing biosynthesis of the plant's hormone and flavonoids.

To compete with the harsh environmental conditions, different defensive, growth promotion and enzymatic biocontrol assay were performed for the selection of the potential biocontrol agent. The importance of protease, siderophore, chitinase and β-1, 3-glucanase in plant defense and growth promotion was reported by (Ganapathi et al., 2008; Dellagi et al., 2009). The observation on enzyme production and growth promotion assay in the isolates, ENF22 (*Penicillium simplicissimum*), ENF31 (*Acremonium* sp.), ENF41 (*Saracladium strictum*) and ENF49 (*Aspergillus ustus*) was found to be promising and could withstand extreme environmental stress conditions. ENF31 was observed with the highest production of protease, 3.66 ± 0.01 mm zone diameter and protease enzyme is a key factor that protect host against wide range of pathogens, including insect pests and nematodes, also fungal protease, At1, is believed to facilitate the cuticular penetration during insect infection and fungal colonization of plants, proving its multifunctional lifestyle (Tunlid et al., 1994; Reddy et al., 1996; Barelli et al., 2016). Root colonization ability is again a major factor that influences the PGP and biocontrol activity, acting as the first line of defense against the root and seed borne phytopathogens. The isolate ENF22 and ENF3, with highest protease activity recorded, were able to colonized maize and rice plant successfully enabling the interaction between endophyte and pathogen that can help us in better understanding and study the overall plant health, growth, and yield improvement. In conclusion, results obtained from the present study encourages us to further investigate on the selected fungal endophytes in order to develop a strong Bio-agent with wide applicability to multi-field and henceforth emerges as a successful bioinoculum leading toward organic food crops for a better tomorrow by reducing the excessive used of chemicals.

## Author contributions

MP: Carried out the research work. SD: PI and mentor of the project. DS: Guided and assisted in manuscript editing GS: Technical guidance in experimental issues and manuscript editing.

### Conflict of interest statement

The authors declare that the research was conducted in the absence of any commercial or financial relationships that could be construed as a potential conflict of interest.
